# Neural Dynamics of Processing Inflectional Morphology: An fMRI Study on Korean Inflected Verbs

**DOI:** 10.3390/brainsci14080752

**Published:** 2024-07-26

**Authors:** Joonwoo Kim, Sangyub Kim, Kichun Nam

**Affiliations:** 1 Department of Psychology, Korea University, Seoul 02841, Republic of Korea; psymon@korea.ac.kr; 2 Department of Psychology, Chonnam National University, Gwangju 61186, Republic of Korea; sangyub0310@jnu.ac.kr

**Keywords:** visual word recognition, inflectional morphology, morphologically rich language, fMRI

## Abstract

The present study aimed to elucidate the neural mechanisms underpinning the visual recognition of morphologically complex verbs in Korean, a morphologically rich, agglutinative language with inherent polymorphemic characteristics. In an fMRI experiment with a lexical decision paradigm, we investigated whether verb inflection types (base, regular, and irregular) are processed through separate mechanisms or a single system. Furthermore, we explored the semantic influence in processing inflectional morphology by manipulating the semantic ambiguity (homonymous vs. unambiguous) of inflected verbs. The results showed equivalent activation levels in the left inferior frontal gyrus for both regular and irregular verbs, challenging the dichotomy between the two. Graded effects of verb regularity were observed in the occipitotemporal regions, with regular inflections eliciting increased activation in the fusiform and lingual gyri. In the middle occipital gyrus, homonyms showed decreased activation relative to that of unambiguous words, specifically for base and irregular forms. Furthermore, the angular gyrus exhibited significant modulation with all verb types, indicating a semantic influence during morphological processing. These findings support single-system theories and the connectionist framework, challenging the assumptions of purely orthographic morphological decomposition and dual-mechanism accounts. Furthermore, they provide evidence for a semantic influence during morphological processing, with differential reliance on semantic activation for regular and irregular inflections.

## 1. Introduction

The issue of how various types of inflection, such as regular (e.g., work—worked) and irregular (e.g., catch—caught) forms, are represented and processed in the brain remains a central topic in psycholinguistics. Classical full-listing models argue that all morphologically complex words are stored in their entirety, regardless of regularity [1]. On the other hand, full-parsing models propose that these forms undergo an obligatory decomposition into their morphemic units, with only morphemes being stored [2,3]. Bridging these two opposing views, dual-mechanism accounts argue that there are categorical differences between regular and irregular forms, suggesting they are processed by distinct mechanisms [4,5,6]. Regular forms, which follow predictable patterns of inflection (e.g., adding ‘–ed’ to form the past tense), are processed through a rule-based system, while irregular forms are stored and processed in the mental lexicon through lexical-semantic memory. In contrast, single-system accounts challenge this dichotomy, suggesting continuous rather than categorical distinctions between regular and irregular inflections [7,8,9]. These theories, based on earlier connectionist models [10,11], advocate for a single associative system that maps orthographic or phonological forms to semantic meanings, leading to graded effects in inflectional morphology.

There has been a heavy focus on the debate surrounding the past tense in English in the previous literature, despite the cross-linguistic differences between writing systems, such as English and Korean. Furthermore, inconsistent findings may derive from these differences, especially in the morphological architecture of languages [12]. Thus, this suggests that the investigation of the processing of morphologically rich languages is crucial for a comprehensive understanding of linguistic structures and cognitive processing across different language systems. As an agglutinative language, Korean is characterized by its highly inflective and polymorphemic nature in which a verb stem is conjugated with a variety of endings to denote variances in tense, mood, and levels of politeness. For example, the stem of the verb *먹다* (to eat) can be conjugated with various endings such as –*습니다* and –*었다* to denote the formal polite present tense *먹습니다* (formed by adding –*습니다* to *먹*–) or the casual past tense*먹었다* (formed by adding –*었다* to *먹*–). Furthermore, irregular verbs in English, while even more frequently used than regular verbs [13,14], follow distinct patterns of vowel and consonant changes or complete suppletion, such as “go—went—gone” and “be—was/were—been” [15]. However, Korean features a highly systematic inflectional morphology in which the majority of verbs are inflected without alternations to the verb stem (e.g., *먹*–) and thus are categorized as regular verbs. Only a subset of ‘irregular’ verbs have accompanying changes in their stems based on consistent and predictable phonological rules [16], such as dropping certain consonants or adding specific vowels. For example, in *ㅂ* irregular verbs, *ㅂ* contained in the verb stem is transformed into a consonant *ㅗ* or *ㅜ* when conjugated with endings starting with the vowels *ㅏ* or *ㅓ*, such as in *도와* (formed by adding –*아* to*돕*–; to help and). 

**Figure 1:**
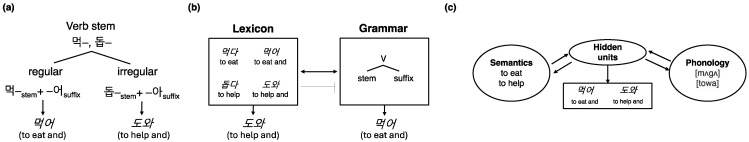
Schematic representation of two opposing views on verb regularity with an example of Korean regular and irregular inflections. (**a**) An example of Korean regular and irregular inflections. (**b**) Schematic representation of the dual-mechanism accounts of inflectional morphology [17]. (**c**) Schematic representation of the single-system theory of morphology [7].

These unique morphological characteristics make the Korean writing system suitable for testing the existing models of morphological processing (Figure 1). At first glance, the full-parsing models may seem plausible for explaining the processing of Korean inflectional morphology, considering its extensive verb conjugation and systematic inflection patterns. However, a potential semantic influence might emerge during the visual recognition of morphologically complex words, which would contradict a purely orthography-based morphological decomposition in a ‘blind-to-semantics’ manner, as has been assumed in these models. Meanwhile, dual-mechanism accounts of inflectional morphology, distinguishing between rule-based regular forms and memory-based irregular forms, may need to be adapted for the Korean writing system, in which even irregular forms follow predictable phonological rules. On the other hand, single-system accounts, which suggest a continuous mapping of forms to meanings, may find support in the consistent and systematic Korean inflectional morphology. In this regard, the present study employed inherently polymorphemic Korean verbs, aiming to investigate if an inflected form elicits a processing cost and whether inflected verbs undergo memory-based retrieval from the mental lexicon and/or are processed via the application of automatic grammar rules.

In the functional magnetic resonance imaging (fMRI) domain, recent studies have investigated brain regions involved in processing morphologically complex words. Initial fMRI research focused on identifying brain regions specifically tuned to morphological processing [18,19]. Devlin et al. [19] observed reduced activation in temporal and parietal regions, such as the bilateral angular gyrus (AG), left occipitotemporal cortex, and left middle temporal gyrus (MTG) for morphologically related word pairs compared to unrelated pairs, suggesting that morphology results from the convergence of form and meaning. Similarly, in an fMRI study using a masked priming paradigm, Gold and Rastle [20] reported the involvement of occipital regions, including the fusiform gyrus (FG) and middle occipital gyrus (MOG), in both morphological and orthographic relationships, with semantic conditions reducing activation in the MTG, indicating the structural nature of early morphological decomposition. The debate continues about whether morphologically complex words are processed as whole units or decomposed into morphemes. Some neuroimaging evidence supports whole-word processing [19,21], while other evidence favors morpheme decomposition [22]. Whole-word processing highlights lexical-semantic effects in widespread bilateral frontotemporal regions, including the MTG, superior temporal gyrus (STG), and inferior frontal gyrus pars orbitalis (IFG, BA 47) [21,23]. Conversely, decomposition emphasizes the role of the posterior left inferior frontal gyrus (LIFG, BA 44/45) [22]. 

The LIFG has been consistently implicated in the literature as a core region for processing inflectional morphology, particularly the past tense in English, demonstrating distinct activation patterns for regular and irregular inflections. Both types show increased activity in temporal and hippocampal regions, including the FG, MTG, and parahippocampal gyrus (PHG). Regular verbs, however, exhibit greater activation in the LIFG, along with additional regions, such as the middle frontal gyrus (MFG), basal ganglia, and cerebellum [24,25,26,27]. This binary distinction aligns with the dual-route theory, positing two memory systems: the procedural memory system for regular inflections and the declarative memory system for irregular ones [6,17,28]. Neuroimaging studies corroborate this theory, showing selective activation for regular inflections in the procedural memory network, characterized by the left-lateralized frontotemporal network, including the LIFG, basal ganglia, and cerebellum [29,30,31].

However, some researchers provide evidence for a single-system theory, which suggests continuous differences in inflection processing [7,32]. Joanisse and Seidenberg [7] found greater activation for regular compared to irregular verbs in the bilateral IFG during a past-tense generation task. They observed that phonologically similar irregular verbs elicited similar activation to regular verbs, while irregular verbs with no phonological similarity showed increased activity. Desai et al. [32] also reported no additional activation for regular inflected verbs compared to irregular verbs when the levels of phonological complexity were matched. These findings suggest that inflectional morphology is influenced by phonological, semantic, and probabilistic factors rather than a binary rule-based system.

Despite the heavy focus on the past tense in English, some neuroimaging studies have also investigated the neural correlates of inflectional morphology employing morphologically rich languages like Finnish, Japanese, and Korean. Finnish studies showed that inflected nouns elicited an increased activation in the LIFG (BA 47), STG (BA 22), MTG (BA 21), and AG (BA 39), indicating that morphological processing involves suffix stripping at the semantic/syntactic level rather than the visual word form level [33,34]. Japanese studies indicated greater activation in the left MTG (BA 21) for verbs compared to nouns, with selective activation for inflected verbs in the LIFG (BA 44/45) [35]. The only fMRI study on Korean inflected verbs to the best of our knowledge was that by Yim et al. [36], which found similar activation patterns for regular and irregular verbs in temporal regions, including the left MTG, MFG, STG, and PHG, suggesting that Korean relies on memory and meaning irrespective of verb regularity, supporting the single-system theory. 

In sum, the previous fMRI literature has highlighted the distinct neural correlates involved in processing morphologically complex words, identifying widespread activation across occipital, temporal, and frontal regions [19,20,21,22,30,37]. Notably, the left frontotemporal regions, particularly the LIFG, have been proposed as the core network (procedural memory network) for the rule-based computation of regular verbs. In contrast, temporal-hippocampal regions are implicated as the declarative memory network for processing irregular verbs, which relies on the retrieval of semantic information, similar to whole-word processing [26,27,29]. However, some researchers have reported no significant difference between regular and irregular verbs after controlling for phonological complexity [7,32] or found equivalent activation for both inflection types in the temporal regions [36]. These findings suggest that verb regularity effects may be gradually modulated by the convergence of orthographic, semantic, and phonological information rather than by categorical differences among inflection types.

With respect to semantic processing, the LIFG has also been highlighted for its role of top-down control over activating and selecting meanings of homonyms [38,39,40]. Other relevant regions include the MTG, AG, and supramarginal gyrus (SMG), associated with accessing and encoding lexical-semantic representations [39,41,42,43,44,45]. For instance, Hoffman and Tamm [38] found that the inferior frontal gyrus (IFG) and posterior middle temporal gyrus (pMTG) play roles in semantic control and representation, respectively. Nevertheless, it remains uncertain whether semantic information is engaged in the processing of inflected verbs, and if so, whether it is processed by a distinct mechanism depending on their regularity.

In the present study, using a rapid event-related fMRI design with a lexical decision task (LDT), we aimed to investigate if different inflection types in Korean verbs show distinct brain activation patterns and how semantic information affects morphological processing. To address the question of whether regular and irregular verbs are processed categorically or continuously, the present study manipulated three inflection types: base, regular, and irregular verbs. This allows us to tackle the issues regarding not only whether inflected forms incur a processing cost compared to uninflected forms [46,47], but also whether the base form (i.e., uninflected form) and the two inflected forms (i.e., regular and irregular inflections) exhibit graded effects or if only the two inflected forms display distinctive differences. Furthermore, the present study examined the interaction between form and meaning by orthogonally manipulating the semantic ambiguity of words, comparing homonymous words with those possessing a single meaning within each type of inflection [46,48].

We hypothesized that the memory-based retrieval of inflected forms would activate widespread bilateral frontotemporal regions, including the MTG, STG, and anterior IFG (BA 47). In contrast, rule-based decomposition was expected to activate the posterior LIFG (BA 44/45). If regular and irregular inflections showed categorical differences, regular inflections would increase left frontotemporal activation, especially in the posterior LIFG. If graded effects were assumed, both inflections would increase activation in the left frontotemporal network, with regular verbs showing the most pronounced activation. Semantic effects were expected to modulate activation in the inferior parietal lobe (IPL), including the AG and SMG.

## 2. Materials and Methods

### 2.1. Participants

Twenty-four healthy native Korean speakers participated in the experiment (12 female; 23.8 ± 2.5 years, *M* ± SD). All had normal or corrected-to-normal vision and were rated as right-handed by the Edinburgh Handedness Inventory [49]. They signed their written informed consent and were compensated with payment for their participation. Two participants’ data were excluded from the further analyses due to their poor behavioral performance, showing accuracy below 70%. The remaining twenty-two participants (11 female) had a mean age of 23.77 ± 2.62 years.

### 2.2. Materials

The experimental stimuli set comprised 240 items, including 120 Korean words and 120 pseudowords, selected from Korean Sejong Corpus [50], which has 15 million words. All word stimuli were verbs with 2–3 syllables, as confirmed by the Standard Korean dictionary. Only pure Korean words were utilized to avoid potential confounding effects of Sino-Korean words (i.e., Chinese-derived words; see [51]). The stimuli set used in the experiment is shown in Appendix A, in which Table A1 and Table A2 represent words and pseudowords, respectively.

The words were categorized into three inflection types, including uninflected base form (*n* = 40), regularly inflected form (*n* = 40), and irregularly inflected form (*n* = 40). As suggested in the previous literature on Korean inflectional morphology [52,53], base forms consisted of a verb stem and a basic ending suffix (–*다*), as in *씻다* (to wash) with the stem *씻*– and suffix –*다*. Regular inflections were formed by the simple addition of a suffix to the verb stem (e.g., *먹어—*“to eat and” with the stem *먹* and the suffix *어*), while irregular inflections involved a transformation of the stem when a suffix was used, as in *도와* (“to help and”) with the stem *돕*– transformed to *도와* when the suffix–*아* was used. 

Semantic ambiguity was manipulated by comparing verbs with single meanings (i.e., control; *n* = 60) and those with multiple unrelated meanings (i.e., homonyms; *n* = 60). The classification was based on objective measures from the given corpus and the Standard Korean dictionary. Subjective measures were also utilized, in which three sets of subjective ratings were conducted on the experimental stimuli: familiarity, the number of meanings (NOM), and the relatedness of meanings (ROM). Twenty volunteers (all native Korean speakers, 13 female; mean age 23.1 years, *SD* = 3.7). For familiarity rating, participants rated the subjective familiarity and frequency of use on a 7-point Likert scale. 

The subjective ratings of the NOM and ROM were carried out following the procedure of Azuma [54]. For the NOM rating task, they indicated whether a presented stimulus had no meaning (0), a single meaning (1), or multiple meanings (2). Stimuli were presented in their base form (e.g., stem suffixed with the basic ending suffix ‘–*다*’), including 60 control stems, 60 homonymous stems, and 60 pseudostems. The base form of pseudostems was created by randomly selecting syllables from real verbs. NOM ratings showed that homonymous stems had significantly more meanings compared to control stems (homonym: *M* = 1.43, *SD* = 0.43; control: *M* = 1.18, *SD* = 0.17; *t*(59) = −6.40, *p* < 0.001). For the ROM rating task, participants judged the degree of relatedness of meanings on a 7-point Likert scale, ranging from 1 (unrelated meanings) to 7 (same meaning). A base form of the stem and exemplar sentences of its meaning pair were presented. For stems with more than two meanings, the most frequently used meaning was paired with another meaning in multiple trials, and the rating score was averaged over all pairwise comparisons. The ROM rating set included 60 homonymous stems and 10 polysemous stems as a filler condition. ROM ratings confirmed that homonymous stems had relatively unrelated meanings (*M* = 1.89, *SD* = 1.50), compared to the highly related meanings of polysemous stems (*M* = 5.35, *SD* = 1.62).

Moreover, the meaning dominance of homonyms was also assessed through additional subjective ratings. Here, twenty native Korean speakers (16 female; mean age 27.7 years, *SD* = 4.59) who did not participate in the main experiment or the three sets of subjective ratings were asked to judge the subjective familiarity of each meaning of the homonyms on a 7-point Likert scale. The results demonstrated no significant difference among conditions in the meaning dominance, showing the mean familiarity ratings for dominant and subordinate meanings: base form homonyms scored 6.12 (*SD* = 0.53) for dominant meanings and 4.54 (*SD* = 1.05) for subordinate meanings; regular form homonyms scored 5.95 (*SD* = 0.52) for dominant meanings and 4.25 (*SD* = 1.07) for subordinate meanings; and irregular form homonyms scored 5.98 (*SD* = 0.46) for dominant meanings and 4.11 (*SD* = 0.94) for subordinate meanings. 

All frequency measures were log-transformed, per million occurrences in the given corpus. The mean log-transformed word frequency per million for each condition was as follows: base control, 1.16 (*SD* = 0.51); base homonym, 0.9 (*SD* = 0.52); regular control, 1.12 (*SD* = 0.57); regular homonym, 1.11 (*SD* = 0.5); irregular control, 1.29 (*SD* = 0.7); and irregular homonym, 1.14 (*SD* = 0.52). The mean log-transformed cumulative stem frequency per million for each condition was as follows: base control, 1.95 (*SD* = 0.45); base homonym, 1.78 (*SD* = 0.56); regular control, 1.67 (*SD* = 0.73); regular homonym, 2.33 (*SD* = 0.61); irregular control, 1.71 (*SD* = 0.37); and irregular homonym, 1.63 (*SD* = 0.71). Other sublexical and lexical variables known to affect behavioral and brain responses during visual word recognition, including word frequency [55], stem length [56,57], stem frequency [58], neighborhood density (i.e., type frequency) [59,60], cumulative frequency (i.e., token frequency) of a first syllable [60,61], and familiarity [62,63] were also statistically matched between experimental conditions (all *p*s > 0.05; see Supplementary Table S1).

Finally, 120 pronounceable pseudowords were created for the filler word condition by randomly selecting and concatenating verb stems and suffixes used in the word stimuli set. The length of stimuli of each condition, including pseudowords, was matched so that half of the stimuli had 2 syllables, and the other half had 3 syllables. 

### 2.3. Procedure

**Figure 2:**
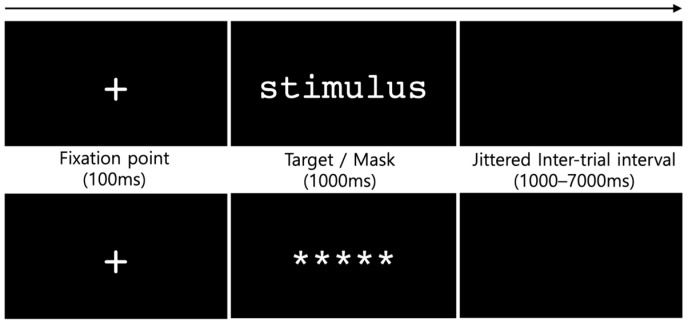
Rapid event-related fMRI paradigm used in the study. The plus sign (‘+’) represents a visual fixation point, and a series of asterisks (‘*****’) denotes a non-linguistic baseline mask.

A rapid event-related fMRI design was employed for the lexical decision task (Figure 2). Each trial commenced with a fixation point (+) displayed for 100 ms, followed by the presentation of a target stimulus for 1000 ms. Participants responded using their index and middle fingers to indicate whether the stimulus was a word or nonword, respectively. The responding hand was counterbalanced across participants: half (*n* = 12) used their right hand, while the other half (*n* = 12) used their left hand. Following each trial, a blank screen was presented during the inter-trial interval (ITI), which varied randomly in duration from 1000 ms to 7000 ms.

All stimuli were presented in white font (Courier New, size 28) on a black background, arranged in a pseudo-random order. The duration of the jittered intervals and the sequence of target stimuli were optimized using optseq2 software [64], and the presentation was controlled with the E-prime 2.0 Professional program (Psychology Software Tools, Inc., Sharpsburg, PA, USA). During the experimental session, stimuli were presented in 4 blocks, each consisting of 60 trials, with a one-minute break between blocks. Prior to the experimental session, the participants completed 30 practice trials. These included 5 baseline masks, 10 words, and 10 pseudowords not part of the experiment session. All participants achieved an accuracy rate above 80% in the practice session, ensuring their understanding of the procedure. 

### 2.4. Image Acquisition

MRI scanning was performed using a Siemens Magnetom Trio 3T MRI scanner (Erlangen, Germany) at the Korea University Brain Imaging Center. Functional images were acquired using T2*-weighted gradient EPI (echo-planar imaging) sequences (TR = 2000 ms; TE = 20 ms; flip angle = 90°; field of view = 240 mm; slice thickness = 3 mm; no gap for 42 slices; matrix size = 80 × 80; and voxel size = 3 mm × 3 mm × 3 mm). T1-weighted structural images were acquired with a 3D MP-RAGE (magnetization-prepared rapid gradient echo) sequence (TR = 1900 ms; TE = 2.52 ms; flip angle = 90°; field of view = 256 ms; matrix size = 256 × 256; and voxel size = 1 mm × 1 mm × 1 mm) covering the whole head.

### 2.5. Behavioral Analysis

A linear mixed effects model (LMM) [65] was used for response time (RT) analysis with lme4 package [66] in R software (version 4.3.1 [67]). Only correct responses were included in the RT analysis. Accuracy data were analyzed with a general linear mixed effects model (GLMM) with a binomial distribution. Both the RT and accuracy analyses included the fixed factors of ambiguity (2: control vs. homonym) and morphology (3: base vs. regular vs. irregular) with random intercepts of both participant and item. Here, *p*-values were calculated using the likelihood ratio test (LRT). For the main effects, a model that included only random factors was compared with a model that incorporated each fixed factor individually. For interactions, *p*-values were calculated by comparing the full model to a model that excluded the interaction term. When any significant effect was found, a post-hoc paired comparison was carried out using estimated marginal means with Bonferroni correction for multiple comparisons. R software [67] was used for the statistical analyses of behavioral and fMRI data.

### 2.6. fMRI Analysis

The fMRI data were processed and analyzed using the SPM12 toolbox (Wellcome Trust Centre for Neuroimaging, London, UK; “http://www.fil.ion.ucl.ac.uk/spm/software/spm12/ (accessed on 1 July 2023)”) alongside custom-built MATLAB scripts [68]. The first three images from each session were discarded to mitigate the transition effects of hemodynamic responses. The remaining functional images underwent realignment for motion correction, followed by slice timing correction to adjust for acquisition time differences between slices. The structural images were then co-registered to the mean functional images and segmented. Subsequently, the functional images were spatially normalized to a standard MNI (Montreal Neurological Institute) template, using the parameters obtained during segmentation. To reduce spatial noise, all images were smoothed with an isotropic Gaussian kernel with a full-width half-maximum (FWHM) of 6 mm.

#### 2.6.1. Statistical Analysis

Statistical analyses were conducted using a general linear model (GLM) with a two-stage mixed effects approach. At the individual level, contrast images between the experimental conditions and baseline masks were generated, resulting in six contrasts: base control > mask, base homonym > mask, regular control > mask, regular homonym > mask, irregular control > mask, and irregular homonym > mask. BOLD signals were convolved with a standard hemodynamic response function (HRF). Only correct responses in the lexical decision task were included in the analysis. Movement parameters obtained during realignment were entered as regressors in the model specification. Additionally, response time (RT) for each condition and participant was included as a parametric modulator, following the method suggested by Taylor et al. [69]. At the group level, one-sample t-tests were conducted on the contrast images estimated at the individual level. A whole-brain statistical parametric map was constructed for each morphology and semantic ambiguity condition. The voxel-level statistical threshold was set at *p* < 0.001, and the cluster-level threshold was set at *q* < 0.05 with false discovery rate (FDR) correction, requiring a minimum cluster extent of 30 contiguous voxels (*k_E_* > 30).

To further investigate the activation patterns observed in the GLM results, follow-up analyses of variance (ANOVAs) were performed with the factors of morphology (3: base vs. regular vs. irregular) and semantic ambiguity (2: control vs. homonym) using beta estimates extracted from significant clusters. Post-hoc pairwise *t*-tests with Bonferroni correction for multiple comparisons were carried out if significant effects were found. 

#### 2.6.2. ROI Analysis

Region of interest (ROI) analyses focused on brain regions have previously been associated with morphological and semantic processing [25,70]. ROIs for morphological processing include the LIFG, specifically the pars triangularis (BA 44/45 [−46, 30, 14]) and pars orbitalis (BA 47 [−37, 31, −12]). Semantic processing regions include the bilateral AG (BA 39; left AG [−45, −61, 36]; right AG [44, −59, 39]) and the MTG (BA 40; left MTG [−56, −34, −2]; right MTG [56, −37, −2]). All ROIs were defined as spheres with a 5 mm radius centered at the corresponding MNI coordinates, selected from the Automatic Anatomical Labeling Atlas 3 (AAL3) [71].

Raw average ROI parameter estimates for each of the six experimental conditions contrasted to baseline masks (e.g., base control > mask) were extracted using the MarsBaR toolbox [72]. Separate 2 × 3 repeated measures ANOVAs were conducted for each ROI with the factors of morphology (base vs. regular vs. irregular) and ambiguity (control vs. homonym), followed by post-hoc pairwise t-tests. Additionally, one-sample *t*-tests were conducted to assess (de)activation of each inflection type relative to the non-linguistic baseline mask (e.g., regular > mask).

## 3. Results

### 3.1. Behavioral Results

The participants’ behavioral performance on the lexical decision task is displayed in Table 1. Trials with reaction times exceeding three SDs (standard deviations) from the mean or with error rates above 30% were excluded from the analysis. This accounted for 9.17% of the responses to the word targets. Only correct responses were included in the RT analysis. The overall accuracy was 90.94 ± 28.72%, and the RT was 580 ± 117 ms on average.

A linear mixed effects model conducted on the RT data revealed the significant effect of morphology (*χ*^2^(2) = 19.81, *p* < 0.001). Specifically, the base form elicited faster responses compared to both the regularly and irregularly inflected forms. Post-hoc pairwise comparisons confirmed that the responses were significantly faster for the base form compared to the regular form (*b* = –4.19, *SE* = 8.67 *p* < 0.001) and the irregular form (*b* = –3.68, *SE* = 8.72 *p* < 0.001). There was no significant difference in RTs between the regular and irregular forms (*b* = 0.467, *SE* = 9.09, *p* > 0.1). No further significant effects were observed in the RT analysis.

An analysis of accuracy also demonstrated the significant effect of morphology (*χ*^2^(2) = 25.14, *p* < 0.001), consistent with the RT analysis. The participants responded more accurately to the base form compared to the regular form (*b* = 5.48, *SE* = 0.23, *p* < 0.001) and the irregular form (*b* = 3.47, *SE* = 0.24, *p* = 0.002). Additionally, the interaction between morphology and ambiguity showed a trend toward significance (*χ*^2^(2) = 5.7, *p* = 0.058). Subsequent post-hoc comparisons revealed a significant ambiguity advantage for the base form, in which the homonyms elicited more accurate responses compared to the control words (*b* = 2.192, *p* = 0.03).

### 3.2. GLM Results

**Figure 3:**
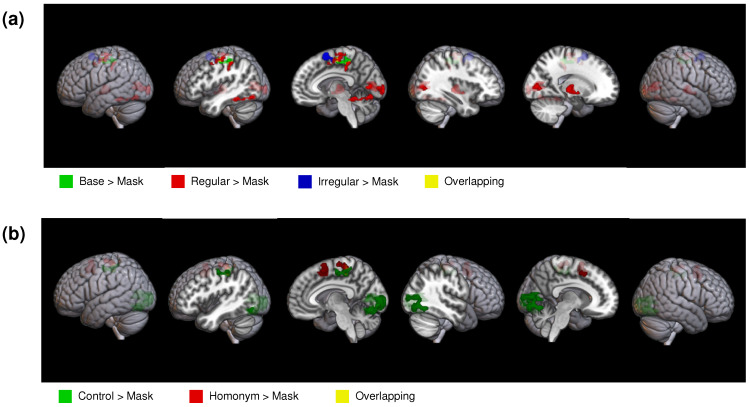
The results of whole-brain analysis for each type of condition contrasted with the baseline mask condition. (**a**) The results of whole-brain analysis for each type of morphology, in which highlighted regions indicate significant effects for base (green), regular (red), and irregular (blue) conditions and overlapping regions (yellow). (**b**) The results of whole-brain analysis for each type of semantic ambiguity condition, in which highlighted regions represent significant effects for control (green) and homonym (red) conditions. The yellow color indicates overlapping regions.

The results of the two-staged mixed effect general linear model (GLM) analysis on the whole-brain activation are detailed in Table 2 and Table 3 and illustrated in Figure 3. A broad array of brain regions associated with language processing showed the activation for each type of morphology and semantic ambiguity condition compared to the non-linguistic baseline mask condition. These regions included the precentral gyrus, thalamus, supplementary motor area, lingual gyrus (LG), MOG, and FG.

**Figure 4:**
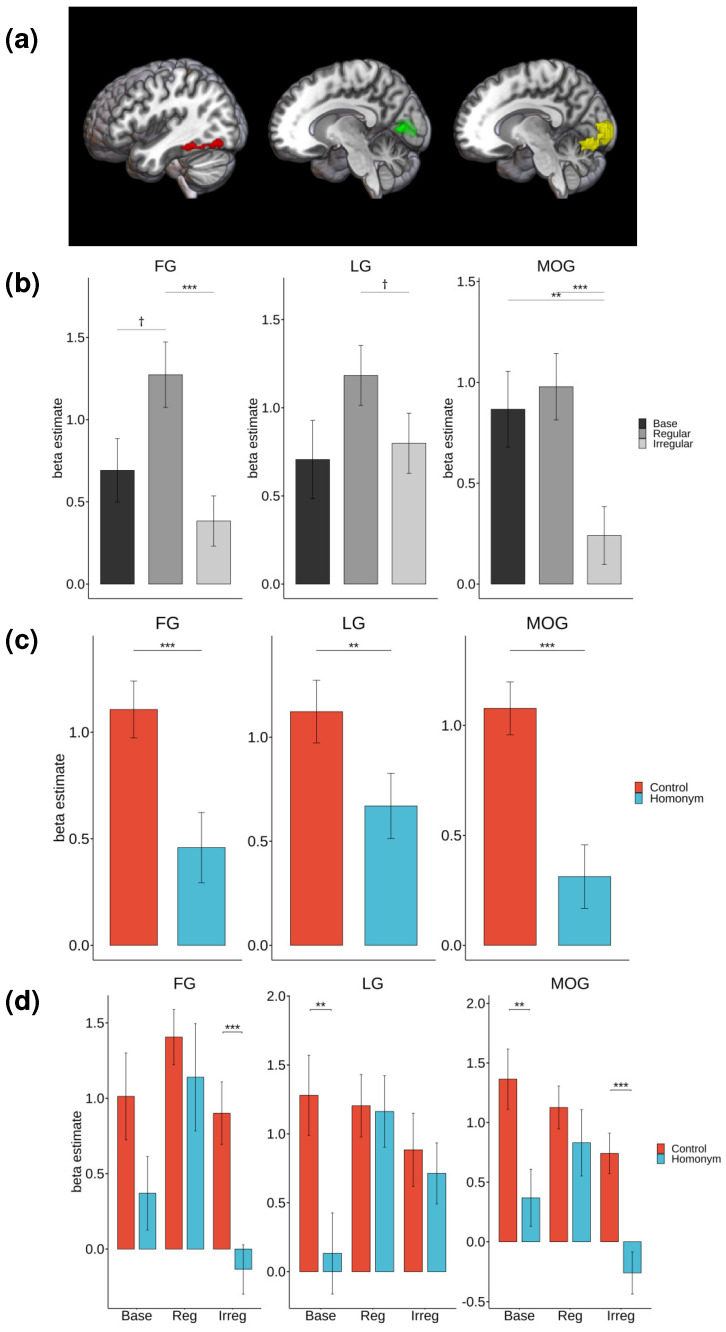
(**a**) The whole-brain activation map of the fusiform gyrus (FG), lingual gyrus (LG), and middle occipital gyrus (MOG). The highlighted regions indicate FG (red), LG (blue), and MOG (yellow). (**b**) Post-hoc analysis results of the main effect of morphology. (**c**) Post-hoc analysis results on the main effect of semantic ambiguity. (**d**) Post-hoc analysis results on the two-way interaction. Note. *** *p* < 0.001, ** *p* < 0.01, † *p* < 0.1.

Follow-up repeated measures ANOVAs were conducted on significant clusters identified in the whole-brain analysis, focusing on occipitotemporal regions including the left FG, lingual gyrus (LG), and middle occipital gyrus (MOG). The factors were morphology (three levels: base, regular, and irregular) and semantic ambiguity (two levels: control and homonym). When significant effects were detected, post-hoc pairwise t-tests with the Bonferroni correction for multiple comparisons were performed, as illustrated in Figure 4.

The main effect of morphology was significant in the FG (*F*(2, 42) = 10.74, *p* < 0.001, *η²* = 0.09) and MOG (*F*(2, 42) = 14.85, *p* < 0.001, *η²* = 0.09) and marginally significant in the LG (*F*(2, 42) = 3.11, *p* = 0.055, *η²* = 0.03). Post-hoc pairwise t-tests revealed that the regular form elicited increased activation in the FG compared to the irregular form (*t*(44) = −4.26, *p* < 0.001). There was also a marginally significant increase in activation for the regular form compared to the base form (*t*(44) = −2.47, *p* = 0.052). In the MOG, there was a significant decrease in activation for irregular forms compared to both the base form (*t*(44) = 3.56, p = 0.003) and the regular form (*t*(44) = −4.58, *p* < 0.001). The LG showed a marginally significant increase in activation for the regular form compared to the irregular form (*t*(44) = −2.34, *p* = 0.071).

The main effect of semantic ambiguity was significant in all of the regions examined. In the FG (*F*(1, 21) = 21.17, *p* < 0.001, *η²* = 0.07), LG (*F*(1, 21) = 10.99, *p* = 0.003, *η²* = 0.03), and MOG (*F*(1, 21) = 33.37, *p* < 0.001, *η²* = 0.13), the homonyms showed reduced activation levels relative to those of control words. The differences were statistically significant with *t*(65) = 3.69, *p* < 0.001 for FG, *t*(65) = 3.00, *p* = 0.004 for LG and *t*(65) = 5.19, *p* < 0.001 for MOG.

The interaction between morphology and semantic ambiguity was significant in the LG (*F*(2, 42) = 6.04, *p* = 0.005, *η²* = 0.04), and marginally significant in the MOG (*F*(2, 42) = 2.47, *p* = 0.096, *η²* = 0.03). An ambiguity advantage, characterized by the increased activation for control words relative to homonyms, was observed in the MOG for both the base form (*t*(21) = 3.53, *p* = 0.002) and the irregular form (*t*(21) = 5.42, *p* < 0.001). In the LG, a significant increase in activation for the control words compared to the homonyms was found for the base form (*t*(21) = 3.81, *p* = 0.001). However, no significant differences between control and homonyms were found in the regular condition for any region (all *p*s > 0.1).

### 3.3. ROI Results

**Figure 5:**
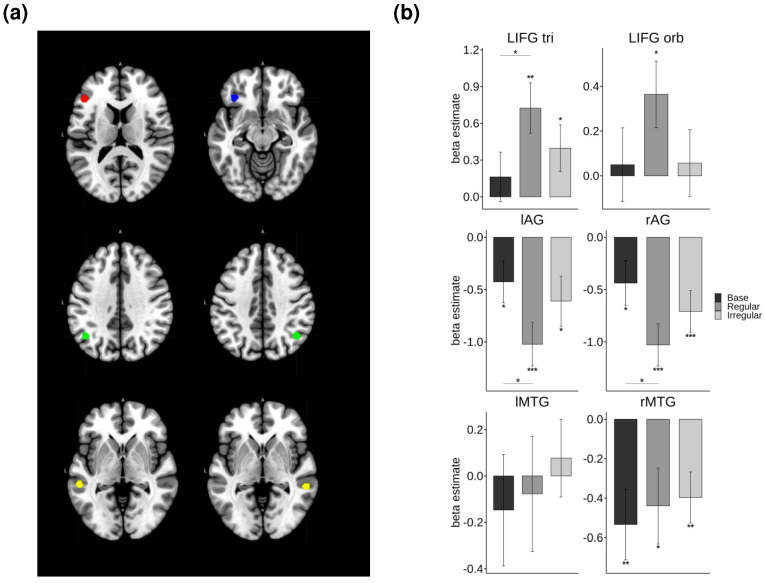
ROI analysis results that showed the significant effect of morphology. (**a**) The regions highlighted in red, blue, green, and yellow represent LIFG pars triangluaris, LIFG pars orbitalis, AG, and MTG, respectively. (**b**) The beta estimates for each condition and statistical comparisons among inflection types. Note. *** *p* < 0.001, ** *p* < 0.01, * *p* < 0.05.

Region of interest (ROI) analyses were conducted on brain regions implicated in the previous fMRI literature [25,70], including the LIFG pars triangularis (LIFG tri) and pars orbitalis (LIFG orb), bilateral AG, and MTG. Figure 5 illustrates the ROIs that showed the significant effect of morphology in the repeated measures ANOVAs, which included the factors of morphology and ambiguity.

Significant effects of morphology were observed in the LIFG tri (*F*(2, 42) = 5.52, *p* = 0.007, *η²* = 0.031) and bilateral AG (left: *F*(2, 46) = 3.83, *p* = 0.03, *η²* = 0.03; right: *F*(2, 46) = 4.17, *p* = 0.02, *η²* = 0.031). The post-hoc paired t-tests indicated a significantly increased activation levels for regular verbs compared to those of base forms in the LIFG tri (*t*(43) = −2.95, *p* = 0.016). Conversely, the regular form showed decreased activation relative to that of the base form in the bilateral AG (left: *t*(43) = 2.91, *p* = 0.006; right: *t*(43) = 2.58, *p* = 0.04).

One-sample t-tests were performed to test for the activation of each inflection type relative to the baseline mask. The results revealed that processing inflected forms (regular and irregular) selectively increased activation in the LIFG tri (regular: *t*(43) = 3.52, *p* = 0.001; irregular: *t*(43) = 2.09, *p* = 0.04), with regular forms also showing increased activation in the LIFG orb (*t*(43) = 2.45, *p* = 0.02). In contrast, processing verbs, regardless of inflection type, was associated with increased deactivation in the left AG (base: *t*(43) = −2.12, *p* = 0.04; regular: *t*(43) = −4.89, *p* < 0.001; irregular: *t*(43) = −2.55, *p* = 0.014) and right AG (base: *t*(43) = −2.05, *p* = 0.046; regular: *t*(43) = −5.12, *p* < 0.001; irregular: *t*(43) = −3.56, *p* = 0.001). A similar pattern of deactivation was also found in the right MTG for all types of verbs (base: *t*(43) = −2.98, *p* = 0.004; regular: *t*(43) = −2.29, *p* = 0.027; irregular: *t*(43) = −3.07, *p* = 0.003).

**Figure 6:**
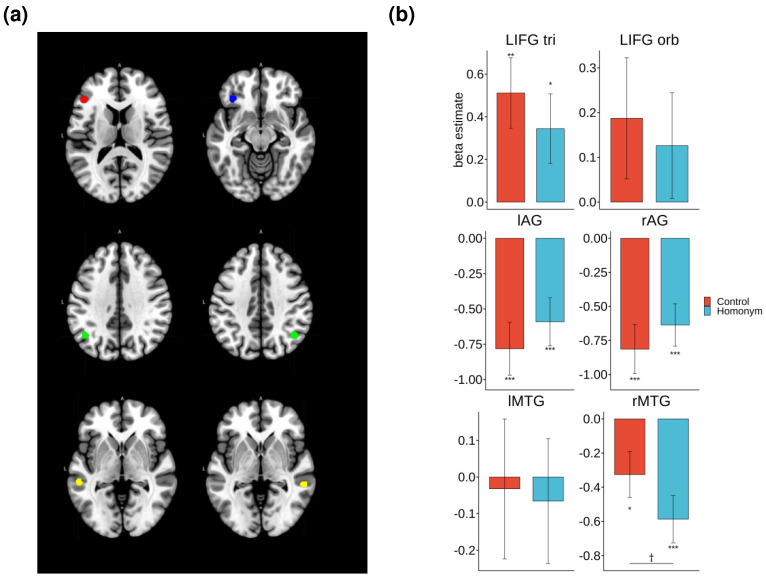
ROI analysis results illustrating the significant effect of ambiguity. (**a**) The corresponding regions highlighted in red (SMG), blue (left MOG), and yellow (right MTG). (**b**) The beta estimates for each ambiguity condition and statistical comparisons between control and homonymous words. Note. *** *p* < 0.001, ** *p* < 0.01, * *p* < 0.05, † *p* < 0.1.

As illustrated in Figure 6, the repeated measures ANOVAs revealed the significant effect of semantic ambiguity in the right MTG (*F*(1, 21) = 5.84, *p* = 0.025, *η²* = 0.014). Specifically, homonyms showed a marginally significant increase in deactivation relative to the control words (*t*(65) = 1.91, *p* = 0.061). No other regions exhibited significant differences in response to semantic ambiguity. A further analysis using one-sample t-tests indicated that homonyms showed a significant deactivation compared to that of the baseline mask in the bilateral AG (left: *t*(65) = −3.49, *p* < 0.001; right: *t*(65) = −4.09, *p* < 0.001) and the right MTG (*t*(65) = −4.25, *p* < 0.001). Similarly, the control words also showed significant deactivation in the same regions (left AG: *t*(65) = −4.16, *p* < 0.001; right AG: *t*(65) = −4.5, *p* < 0.001; right MTG: *t*(65) = −2.42, *p* = 0.018). Importantly, the LIFG displayed the opposite pattern of activation. Both the homonymous and control words elicited increased activation in the LIFG pars triangularis (LIFG tri) (control: *t*(65) = 3.09, *p* = 0.002; homonym: *t*(65) = 2.11, *p* = 0.039).

## 4. Discussion

The present study aimed to investigate the spatial localization of morphological processing using a rapid event-related fMRI design combined with a lexical decision task. The experiment explored how morphological inflection (base, regular, and irregular) and semantic ambiguity (control vs. homonymous) influence both behavioral performance and brain activation patterns during the visual recognition of morphologically complex Korean verbs. It was hypothesized that verb regularity and inflectional cost would be reflected in differential activation in the left frontotemporal regions, particularly the LIFG, with regularly inflected verbs showing increased activity relative to that of base or irregular forms. An interaction between form and meaning was also anticipated, with regularly inflected homonyms expected to show increased activation relative to that of control words in temporal regions, while base and irregular forms were expected to elicit the reverse pattern.

The behavioral results revealed a significant inflectional cost in terms of reaction times and accuracy, indicating that the participants responded faster and more accurately to base forms compared to both regular and irregular inflected forms. Additionally, a significant interaction was observed between morphology and ambiguity. The base forms exhibited an ambiguity advantage, whereas the regular forms displayed a trend towards an ambiguity disadvantage. These findings align with previous research on inflected words in morphologically rich languages, suggesting that uninflected forms are processed more efficiently due to their direct access to semantic representations, while inflected forms require additional processing effort, leading to an increased processing time and error rates [33,73]. Furthermore, the significant interaction between form and meaning suggests that the processing of homonymous words can be facilitated or impeded depending on their morphological form, potentially due to the interplay between morphological parsing and access to semantic representations [74].

The whole-brain fMRI results revealed activations predominantly in occipitotemporal regions associated with morphology and semantics, including the FG, LG, and MOG. A significant modulation of activation by inflection type was observed, with regular forms showing increased activation in the FG and LG compared to that of the base and irregular forms. In contrast, irregular forms exhibited significantly reduced activation in the MOG relative to that of the base and regular forms. These findings are consistent with previous studies implicating the occipitotemporal cortex in visual word form processing and morphological analysis [20,75]. 

The occipitotemporal cortex, especially the visual word form area (VWFA) located within the left FG, has been consistently associated with orthographic processing during the visual recognition of words [20,76,77,78] and thus is often suggested as evidence for a morphological decomposition driven by orthography in a blind-to-semantics manner. For instance, in a masked priming fMRI experiment, Gold and Rastle [14] found an overlapping activation in occipitotemporal regions, including the left fusiform and lingual gyri for pseudo-morphological (e.g., corner—CORN) and orthographic (e.g., brothel—BROTH) conditions, but not for the semantic (bucket—PAIL) condition. Related to this and more importantly, they found a distinctive selective activation of the LG (BA 19) for morphological word pairs compared to that of unrelated pairs, suggesting early morphological decomposition is purely driven by orthography. Indeed, the current data demonstrate the contribution of the FG, LG, and MOG to morphological effects, though with regular forms showing increased activation compared to that of base or irregular forms, challenging the notion of obligatory segmentation processes for all morphologically complex words [2,79].

Furthermore, semantic effects were observed in occipitotemporal regions, with homonyms showing decreased activation relative to that of unambiguous words. A form-with-meaning interaction was found, in which decreased MOG activation for homonyms relative to unambiguous words was evident for base and irregular forms, but not for regular forms. This pattern is consistent with behavioral findings showing a reversal of the semantic ambiguity effect for base and regular forms. The FG’s role in lexical-semantic processing, as suggested by the previous literature [75,80], fits well with the current data, which demonstrate both morphological and semantic effects, indicating the occipitotemporal region’s role in interfacing form and meaning in morphologically complex Korean verbs.

Our ROI analyses, based on the previous fMRI literature [25,70], further elucidate the neural substrates underlying the processing of different morphological and semantic properties of verbs. The analyses focused on the LIFG pars triangularis and pars orbitalis, bilateral AG, and MTG. A significant main effect of morphology was observed in the LIFG tri and bilateral AG. Regular forms showed increased activation in the LIFG tri compared to base forms, while the regular form showed decreased activation relative to the base form in the bilateral AG. The one-sample t-tests indicated that processing inflected forms (regular and irregular) selectively increased LIFG activation in the LIFG tri, with regular forms also showing increased activation in the LIFG orb. In contrast, all verb types, regardless of inflection, were associated with increased deactivation in the bilateral AG and right MTG.

In the dual-route model of inflectional morphology [6,17,28], the declarative memory network involving the temporal-hippocampal regions is expected to modulate both types of inflection. In contrast, the rule-based processing of regularly inflected verbs is associated with distinctive activation in the procedural memory network, which includes the LIFG, cerebellum, and basal ganglia. According to this model, regular inflections should uniquely engage the LIFG and related structures, while both regular and irregular inflections should activate the temporal-hippocampal network. We indeed observed a significant reduction in AG activation for all verb types compared to the baseline mask, with the regular inflections showing reduced activity relative to that of base forms. Given the functional association of AG and the inferior parietal lobule (IPL) with semantic processing [70,81], these results suggest that the processing of base form verbs benefited more from memory-based retrieval processes relative to regularly inflected verbs and that these processes are involved in processing Korean verbs, regardless of whether they are inflected, and if so, whether they are regularly or irregularly inflected. 

Contrary to the expectation of the dual-route model, the posterior division of the LIFG (i.e., LIFG pars triangularis, BA 44) showed selective activation for both inflected forms but not for uninflected base forms. Importantly, regular and irregular verbs did not differ significantly in LIFG activation, with irregular verbs eliciting equivalent levels of activation, challenging the binary distinction of LIFG activation patterns for regular and irregular inflections. Furthermore, the current results contradict the notion that all morphologically complex words are processed as whole words, which would predict similar activation levels for regular and irregular inflections across widespread bilateral regions, including the IFG pars orbitalis, MTG, and occipitotemporal regions. Instead, the present findings support a single-system mechanism by which continuous rather than categorical differences between regular and irregular inflections are expected. These results align with a more general function of the LIFG, which involves processing morphologically complex words, as suggested by the previous fMRI literature on derivation and inflection [22,26,33,34].

Furthermore, semantic effects were reflected in the modulation of the temporal region, particularly the right MTG, along with the occipitotemporal regions observed in the whole-brain results. The functional contribution of temporal regions to semantic processing is well documented [41,42]. Surprisingly, increased activation in these regions, interpreted as engaging greater neural resources to resolve semantic ambiguity in previous research [39], contradicts the behavioral findings of this study, in which facilitative effects for homonyms were observed for base and irregular forms. However, a significant difference in right MTG activation between homonymous and unambiguous words suggests that facilitative effects for homonyms may be due to greater activation of abundant lexical-semantic representations [82,83]. Furthermore, both homonymous and unambiguous words elicited equivalent activation in the LIFG pars triangularis, suggesting that the processing of homonyms relies more on lexical-semantic representations than on the top-down regulation of multiple competitors.

Finally, the current findings in Korean, a morphologically rich and agglutinative language, underscore the importance of exploring morphological processing in languages beyond the widely studied Indo-European family. Similar research typically focuses on languages with inflectional suffixes, yet it remains unclear how our methodology might apply to languages that utilize prefixes or exhibit mixed morphology. Languages with prefixes, such as Swahili, and those with mixed morphological systems, like German, may process morphological structures differently due to their unique combinatory patterns of morphemes. Future research should consider adapting our approach to investigate whether similar neural mechanisms are involved in these languages. 

Moreover, the languages considered in our study are strongly agglutinative, characterized by clear and regular morpheme boundaries. This raises the question of whether our findings would hold true in languages with a higher degree of synthetic inflection, which generally exhibit more irregular and fusional morphologies. Languages such as Latin or Russian, which often blend morphemes into more complex forms, could potentially demonstrate different patterns of neural activation due to the increased cognitive demands of parsing these forms. Investigating such languages could reveal whether the graded effects of verb regularity and the semantic influence observed in Korean are universally applicable or specific to agglutinative languages.

## 5. Conclusions

The present study investigated the spatial dynamics underlying the visual recognition of morphologically complex Korean verbs using a rapid event-related fMRI design. The findings challenge the dual-route model of inflectional morphology [6,17,28], which posits distinct processing pathways for regular and irregular verbs. Contrary to this model, both regular and irregular verbs showed equivalent levels of activation in the LIFG, a core region in the proposed procedural memory network. Instead, these results support the general role of the LIFG in handling morphological processing demands, a role primarily demonstrated in studies of morphologically rich languages [33,73]. Importantly, graded effects for verb regularity were observed in the bilateral occipitotemporal regions, with regular inflections eliciting increased activation in the left FG compared to base and irregular forms. The AG, a region in the IPL consistently associated with semantic processing, was differentiated between the three inflection types, with the base forms showing the lowest levels of deactivation, followed by the regular and then irregular inflections. These results provide evidence for the single-system mechanism [7,32], which argues for a continuous effect of verb regularity, with orthographic, semantic, and phonological properties converging in the processing of words. Furthermore, the facilitative effect of semantic ambiguity was reflected in the selective deactivation of the MTG and MOG for homonyms, while the LIFG showed equivalent activation for both homonymous and unambiguous words. These findings suggest that processing Korean homonyms may benefit from facilitated access to lexical-semantic information rather than being inhibited by the processes of selection and regulation among multiple meanings [84,85].

The pattern of activation observed across widespread bilateral inferior frontal, temporal, and occipitotemporal regions for processing morphologically complex Korean verbs suggests that the integration of morphological and semantic information involves a complex network of brain regions. These results align with the view that posits a distributed function involving extensive neural networks involved in morphological processing [86] and, more generally, in language processing [87,88]. 

## Figures and Tables

**Table 1:** Mean reaction time (RT, in ms), percentage of accuracy (ACC, %), and standard deviations (SD, in parenthesis) for six conditions.

Inflection Type	Semantic Ambiguity			
	Control		Homonym	
	RT (ms)	ACC (%)	RT (ms)	ACC (%)
Base	569 (113)	93.38 (24.89)	555 (105)	96.78 (17.67)
Regular	583 (114)	87.98 (32.56)	602 (122)	85.19 (35.58)
Irregular	588 (122)	91.85 (28.87)	594 (121)	90.12 (29.87)

Note. Reaction times (RTs) for correct responses only.

**Table 2:** Brain regions that showed significant effect for each type of morphological condition, relative to the baseline mask condition.

Contrast	Cluster Size	Brain Regions	Hemisphere	MNI Coordinates			Z
				x	y	z	
Base > Mask	89	Precentral gyrus	L	−36	−28	56	5.75
Reg > Mask	71	Calcarine	L	−12	−100	−4	5.10
	32	Middle occipital gyrus	R	36	−76	2	3.40
	116	Calcarine	R	9	−82	8	4.60
		Lingual gyrus	L	0	−73	5	3.97
		Calcarine	R	6	−88	2	3.94
	55	Postcentral gyrus	L	−48	−16	56	4.54
		Precentral gyrus	L	−33	−10	65	4.00
				−51	−4	50	3.81
	98	Thalamus	R	21	−22	2	4.23
		Putamen	R	30	−4	−7	3.87
	65	Fusiform gyrus	L	−42	−40	−19	4.16
				−39	−76	−13	3.91
				−42	−55	−19	3.55
	39	Inferior parietal gyrus	L	−39	−28	38	3.98
		Postcentral gyrus	L	−42	−34	50	3.85
Irreg > Mask	55	Supplementary motor area	L	−3	8	65	3.64
		Middle cingulate cortex	L	−3	−4	50	3.44

Note. All regions were significant to *q* < 0.05 FDR-corrected, *k_E_* ≥ 30 at the cluster level, and *p* < 0.001 at a voxel level. Reg = regular inflection; Irreg = irregular inflection.

**Table 3:** Brain regions that showed significant effect for each type of semantic ambiguity condition, relative to the mask condition.

Contrast	Cluster Size	Brain Regions	Hemisphere	MNI Coordinates			Z
				x	y	z	
Con > Mask	285	Calcarine	L	−12	−100	−4	5.18
		Fusiform gyrus	L	−36	−79	−16	4.67
		Middle occipital gyrus	L	−27	−88	2	4.65
	105	Postcentral gyrus	L	−36	−34	56	4.68
			L	−42	−16	50	3.53
		Precentral gyrus	L	−33	−16	50	4.27
	316	Calcarine	R	9	−82	8	4.26
		Lingual gyrus	R	15	−94	−7	4.23
Hom > Mask	119	Supplementary motor area	R	9	14	47	4.55
			L	−6	5	56	4.42
	57	Postcentral gyrus	L	−36	−34	56	4.10
		Precentral gyrus	L	−24	−19	68	3.46
				−33	−22	65	3.46

Note. All regions were significant to *q* < 0.05 at the FDR-corrected level, *k_E_* ≥ 30 at the cluster level, and *p* < 0.001 at a voxel level. Con = Control; Hom = Homonym.

## Data Availability

The data presented in this study are available on request from the corresponding author due to privacy and ethical restrictions, as they contain information that could compromise the privacy of research participants.

## Supplementary Materials

The following supporting information can be downloaded at: https://www.mdpi.com/article/10.3390/brainsci14080752/s1, Table S1: Experimental conditions and statistical comparisons of sublexical and lexical variables.

## Appendix A

Table A1 and Table A2 show the stimuli set used in the experiment, representing words and pseudowords, respectively. The words are categorized under six different conditions: Base Control, Base Homonym, Regular Control, Regular Homonym, Irregular Control, and Irregular Homonym.

**Table A1:** The word stimuli set used in the experiment.

Base Control	Base Homonym	Regular Control	Regular Homonym	Irregular Control	Irregular Homonym
씻다	갈다	죄어	감아	눠서	갈려
신다	깨다	찧어	걷어	썩혀	쳤어
끊다	들다	엮고	개서	기워	쪼여
좇다	뛰다	볶아	불지	붐벼	텄다
놀다	뜨다	꽂고	붓지	모셔	맞혀
낫다	맞다	믿니	빌지	는다	말려
뽑다	맡다	서라	빨면	도와	배겨
돌다	매다	맺자	새자	실어	걷혀
참다	묻다	뚫어	켜며	굽혔다	멘다
씹다	받다	갚게	패지	놓쳤다	져도
찢다	쉬다	빚고	꾸어	어는가	졸려
얻다	타다	넣겠다	굽지	판다고	훔쳐
견디다	구르다	휘었고	거르고	저었고	감겼고
살피다	구하다	굴리지	배어도	뒀다	들려서
버티다	그리다	우기지	끼치고	뭉쳐서	바랬지
벌이다	달리다	감싸면	놀리자	박혔다	물려서
싸우다	따르다	다듬고	닥치지	치민다	써가며
부르다	부치다	넓히며	돋는다	샀고	일렀다
거닐다	빠지다	찾지만	싸놓고	꿴다	익혀서
다투다	울리다	닿았고	불리지	줬고	태웠다

**Table A2:** The pseudoword stimuli set used in the experiment.

Pseudowords					
닥든	멜쳐	폐진	혈긴	치오든	기까래
긴들	어빌	출다	칫자	싸치곤	잡우는
애건	벽다	치펴	드잘	기휠지	설기건
바면	새룬	먹낼	펴잰	먹얹네	꺼담네
버닌	망갠	돛던	휘슨	이패든	뜯리던
취랜	다길	둘긴	다쉴	치린지	싸눌은
제깐	규며	슬신	시딘	까리니	지패게
곰다	푹인	취든	잠선	기힐지	암섰지
태려	채셔	키룬	잴출	씌희네	밑절까
네칠	석린	핏지	줄핀	우이어	높리던
펄다	쨉던	추랜	포실	졸켜니	치철지
몫다	닻다	급던	묵진	끼진가	뜨러니
꾀실	겅틸	둡는	으인다	기묻냐	닫내는
죄릴	귤던	찰댄	주갈라	씌지곤	먹우네
후나	우알	치핀	치놀까	골길까	기말지
껀든	덤네	팝자	키르냐	바몰면	꿰루곤
보난	긷서	매몬	박낸들	꼽운다	차물다
철다	지뺀	삽아	움나며	곯기든	가먹은
빗셔	달탄	볍자	살건다	설까던	팝는다
유길	유날	벗친	보힌대	기훔다	펴려요
