# Supplementary material for: Neural Dynamics of Processing Inflectional Morphology: An fMRI Study on Korean Inflected Verbs
Source: Brain Sci. 2024 Jul 26;14(8):752. doi: 10.3390/brainsci14080752 (PMC11352235; doi:10.3390/brainsci14080752)
Supplement: Supplementary file 1 [file brainsci-14-00752-s001.zip › brainsci-3100598-supplementary.pdf]

Table S1. Experimental conditions and statistical comparisons of sublexical and lexical variables.

Semantic ambiguity	Inflection type	Word Len	Word Freq	Stem Len	Stem Freq	Type Freq	Token Freq	Fam	Obj NOM	Subj NOM	Subj ROM	Subj DOM
Control	Base	2.4 (0.5)	1.16 (0.51)	1.40 (0.50)	3.13 (0. 45)	3.1 (0.68)	3.92 (0.76)	6.24 (1.23)	1 (0)	1.21 (0.41)	-	-
	Regular	2.47 (0.51)	1.12 (0.57)	1.25 (0.44)	2.95 (0.56)	2.78 (0.69)	3.61 (0.79)	5.77 (1.48)	1 (0)	1.20 (0.43)	-	-
	Irregular	2.44 (0.51)	1.29 (0.7)	1.40 (0.50)	2.84 (0.73)	2.97 (1)	3.7 (1.15)	5.74 (1.57)	1 (0)	1.13 (0.42)	-	-
Homonym	Base	2.35 (0.49)	0.9 (0.52)	1.40 (0.50)	3.5 (0.61)	3.14 (0.82)	4.01 (0.92)	6.24 (1.16)	2.13 (0.63)	1.48 (0.51)	1.90 (1.49)	1.58 (0.97)
	Regular	2.4 (0.5)	1.11 (0.5)	1.25 (0.44)	2.89 (0.37)	3.13 (0.61)	3.87 (0.66)	5.88 (1.41)	2.25 (0.44)	1.42 (0.54)	1.92 (1.59)	1.7 (0.91)
	Irregular	2.4 (0.5)	1.14 (0.52)	1.70 (0.47)	2.81 (0.71)	3.03 (0.78)	3.8 (0.96)	5.80 (1.36)	2.55 (0.83)	1.41 (0.50)	1.85 (1.43)	1.87 (0.88)
Statistics		*n.s.* $F = 0.22$, $p = 0.64$	*n.s.* $F = 0.97$, $p = 0.33$	*n.s.* $F = 2.69$ $p = 0.1$	*n.s.* $F = 0.22$, $p = 0.47$	*n.s.* $F = 0.002$, $p = 0.97$	*n.s.* $F = 0.04$, $p = 0.85$	*n.s.* $F = 2.37$, $p = 0.127$	$F = 84.4$, $p < 0.001$	$F = 6.2$, $p = 0.01$	*n.s.* $F = 0.22$, $p = 0.641$	*n.s.* $F = 1.01$, $p = 0.319$

Note. All frequency measures were log-transformed, per million occurrences in the given corpus. *n.s.* = not significant.
Word Len = Word length in syllable; Word Freq = word frequency;
Stem Len = stem length in syllable; Stem Freq = cumulative stem frequency;
Type Freq = type frequency of the first syllable; Token Freq = token frequency of the first syllable;
Subj Fam = subjective word familiarity; Obj NOM = number of objective meanings;
Subj NOM = number of subjective meanings; Subj ROM = subjective relatedness of meanings;
Subj DOM = subjective meaning dominance.
